# Evaluation of Abstraction Capabilities and Detection of Discomfort with a Newscaster Chatbot for Entertaining Elderly Users

**DOI:** 10.3390/s21165515

**Published:** 2021-08-17

**Authors:** Francisco de Arriba-Pérez, Silvia García-Méndez, Francisco J. González-Castaño, Enrique Costa-Montenegro

**Affiliations:** Information Technologies Group, atlanTTic, Telecommunications Engineering School, Campus as Lagoas-Marcosende, University of Vigo, 36310 Vigo, Spain; farriba@gti.uvigo.es (F.d.A.-P.); sgarcia@gti.uvigo.es (S.G.-M.); kike@gti.uvigo.es (E.C.-M.)

**Keywords:** active and healthy ageing, artificial intelligence, human computer interaction, intelligent conversational system, machine learning, natural language processing, robotics and intelligent systems, smart living application

## Abstract

We recently proposed a novel intelligent newscaster chatbot for digital inclusion. Its controlled dialogue stages (consisting of sequences of questions that are generated with hybrid Natural Language Generation techniques based on the content) support entertaining personalisation, where user interest is estimated by analysing the sentiment of his/her answers. A differential feature of our approach is its automatic and transparent monitoring of the abstraction skills of the target users. In this work we improve the chatbot by introducing enhanced monitoring metrics based on the distance of the user responses to an accurate characterisation of the news content. We then evaluate abstraction capabilities depending on user sentiment about the news and propose a Machine Learning model to detect users that experience discomfort with precision, recall, F1 and accuracy levels over 80%.

## 1. Introduction

According to the 2019 Revision of World Population Prospects by the United Nations (available at https://population.un.org/wpp, accessed on 15 August 2021), elderly people (>65 years old) will double by 2050. The population in their eighties or older will reach 450 million people worldwide by that year. Social interaction is a basic need of our ageing society. Solitude can be alleviated with social media [1], but the digital divide is a barrier for non-digital elderly users [2]. This includes those with technological background that lose their cognitive capabilities to access content of their interest, that is, their abstraction skills, for instance at the early stages of cognitive disorders.

In this context, conversational assistant technology will experience a 37% Compound Annual Growth Rate (CAGR) by 2023 (2021 Chatbots Market Research Report, available at https://www.marketresearchfuture.com/reports/chatbots-market-2981, accessed on 15 August 2021). Despite the many technological advances in Artificial Intelligence (AI) for Socially Assistive Robotics (SAR), still more work must be conducted to achieve a practical caregiver chatbot for end users [3]. Instead of pursuing that long-term goal, we have proposed a feasible, realistic approach to an intelligent conversational assistant with video and audio sensors that controls a newscaster, which seeks both to entertain elderly users and augment their abstraction skills [4].

Previous research has noted the low level of engagement of the elderly population with new technologies [5]. This has motivated us to follow an elderly user-centred approach for personalised entertainment content access.

Existing popular voice assistants, such as Siri (available at https://www.apple.com/siri, accessed on 15 August 2021) by Apple, Cortana (available at https://www.microsoft.com/en-us/cortana, accessed on 15 August 2021) by Microsoft, Google Assistant (available at https://assistant.google.com, accessed on 15 August 2021) and Alexa (available at https://developer.amazon.com/alexa, accessed on 15 August 2021) by Amazon, help users find multimedia content and support hands-free control of home devices. However, the technological background they demand [6] is excessive for the elders, who are not interested in these devices as mere toys. They prefer familiar media, such as television or radio broadcasts [4,7]. Accordingly, we seek to augment this type of media with assistant chatbots for enabling intelligent newscasters.

Specifically, we insert short dialogues between newscasts about their content, and we rely on Sentiment Analysis (SA) to automatically adapt the conversation to the users’ mood, so as to emphatically increase the feeling of companionship of traditional media [8]. At the same time, when the data gathered from user answers carry a positive sentiment, they allow extracting valuable insights on user preferences, as well as assessing discomfort if the sentiment is negative. Finally, our conversational system allows extracting metrics about the abstraction skills of the elders. In this work we show that these metrics are strongly dependent on the sentiment of the answers and the level of discomfort.

The rest of this paper is organised as follows. Section 2 reviews related work and highlights our contributions. Section 3 describes our solution for entertaining and transparently monitoring elderly people by extracting their preferences and evaluating their abstraction skills. Section 4 presents our tests with real users from “Asociación de Familiares de Enfermos de Alzheimer y otras Demencias de Galicia” (AFAGA, the Galician Association of Relatives of Patients with Alzheimer’s and other Dementias, available at https://afaga.com, accessed on 15 August 2021). Finally, Section 5 concludes the paper and proposes some future work directions.

## 2. Related Work

State-of-the-art AI and Natural Language Processing (NLP)-boosted conversational assistants can automatically sustain dialogues with end users [9,10,11]. Nowadays, chatbots are commonly applied to management [12,13,14], education [15,16,17] and healthcare [18,19,20]. It has been demonstrated that they perform better when designed on purpose for specific domains and user groups, and when their utterances are short and accurate within those domains. This engages users in longer conversations [21] and creates a feeling of companionship [22].

More in detail, conversational systems can be retrieval-oriented or generative. Retrieval-oriented systems rely on fixed linguistic rules and patterns. This is the case of the well-known Artificial Intelligence Markup Language (AIML) technology, which is widely used to create chatbots [12,23]. However, this approach has partial understanding and lacks the capabilities to augment the dialogue flow from context information [24]. Conversely, generative systems exploit more sophisticated techniques, such as NLP [25] and Machine Learning (ML) [26]. They are more flexible and have the potential to sustain human-like conversations. In light of this, we combined AIML technology with Natural Language Generation (NLG) to enrich the conversational capabilities of the system. Our research belongs to the SAR field [27,28]. It seeks to assist the target users to improve their abstraction capabilities so as to access digital media of their interest and monitor these capabilities, which is a novel approach in gerontology.

We remark that, unlike our approach, existing entertainment chatbots [29,30,31] have not been created with the same target population in mind. Conversational systems for elderly people are typically therapy-oriented [32]. They often require the participation of a caregiver. Thus, they set aside long-term engagement, which we seek with a personalised newscaster to entertain elderly people. A possible exception is RobAlz [33], a storyteller chatbot for elderly people with mild cognitive impairments, but the authors followed a retrieval model based on templates and, therefore, the adaptability of their system to user moods and preferences, as well as its capabilities to automatically gather new content, are limited. Conversely, we apply a generative NLG model that creates coherent, varied short dialogues from the news content and the user context (that is, primarily his/her answers, but also the output of video and audio sensors) to foster engagement and a feeling of companionship.

Regarding user monitoring, we can cite the Ryan robot for cognitive behavioural therapy for elders diagnosed with depressive disorder [34] and the chatbot in [35], which reminds of appointments and medication doses to elderly people, and transmits physiological parameters back to a control centre. However, these have no entertainment capabilities. Besides, their conversational models are limited compared to our generative approach. This is also the case of the NAO [36] entertainment chatbot, which can tell jokes and play music.

Regarding empathetic capabilities, SA algorithms estimate the polarity of textual content, this is, its negative, neutral or positive meaning. They have been extensively used in many application domains, of which business [37] and sociology [38] are very representative. The chatbot in [39] applies both NLP techniques and SA to psychiatric counselling. The empathetic capabilities of the conversational assistant in [40] are very limited due to the general knowledge bases used to generate the utterances, since contextual information was not considered.

A recent proposal of a health chatbot with SA capabilities is described in [41]. It relies on an LSTM–RNN classifier that has been trained with data from Twitter and Reddit. Additionally, the NESTORE e-coach conversational system in [42] has a SA–NLP pipeline based on a formal semantic model. It produces a summary of the emotional state of the users from their responses to template-based messages extracted from an ontology. Unlike our proposal, none of these works use NLG.

As in [39], we combine SA and NLG techniques to adapt the dialogue flow to the users’ mood, yet in a different application domain. Note that our proposal uses SA for adapting both the dialogue flow and the facial expression of the avatar of the conversational assistant, a typical approach in robotic empathy [43].

Ultimately, our research contributes to the state of the art in user-centred expert systems to close the digital gap in our ageing society. Our novel intelligent assistant with video and audio sensors extracts user preferences about newscasts from short, empathetic dialogue stages on the content, and helps users express their interests. In this work we evaluate the capabilities of this assistant to measure the abstraction skills and the discomfort of the users depending on the detected sentiment.

## 3. System Architecture

**Figure 1:**
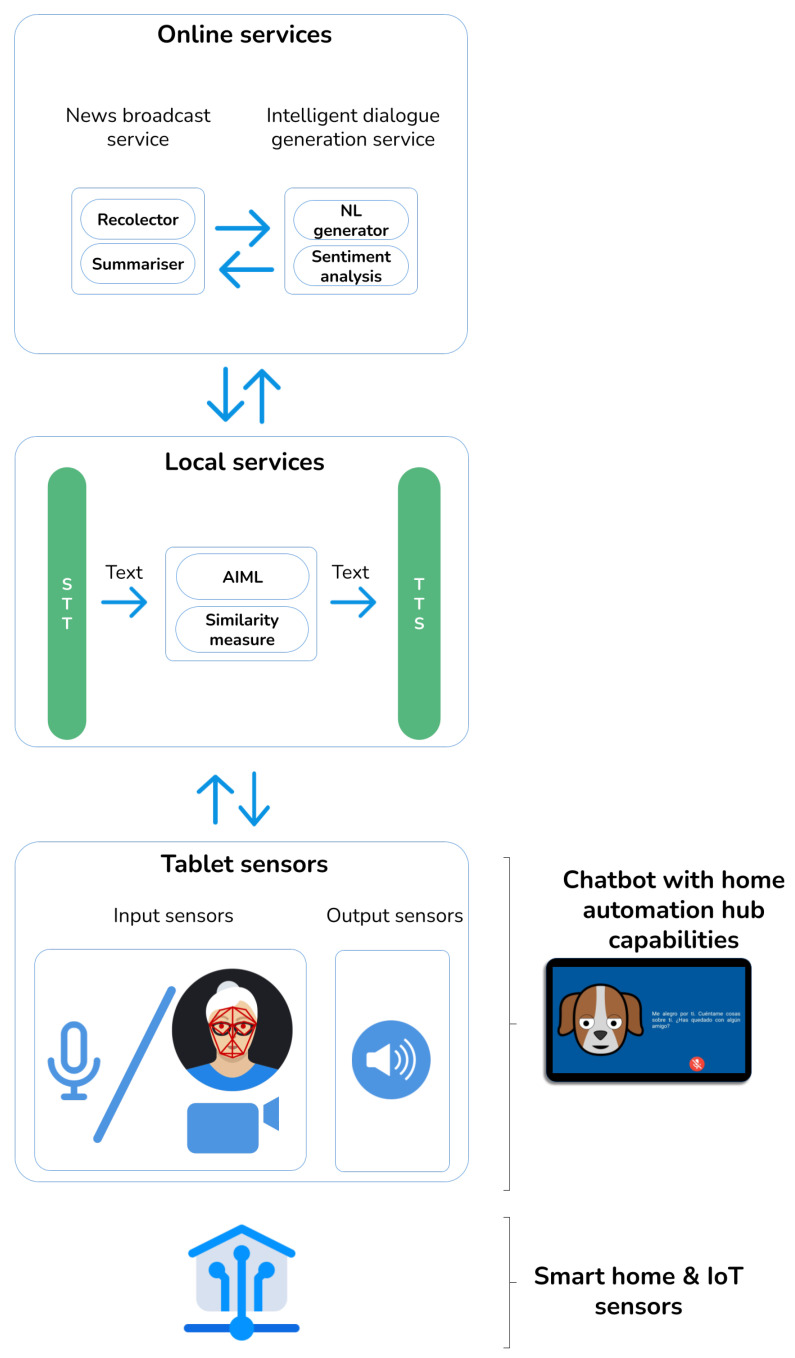
System architecture. STT: speech-to-text conversion, TTS: text-to-speech conversion.

In this section we describe the three-layer architecture of our system (Figure 1), which supports the analysis in Section 4. It is composed of online and local services, such as input/output sensors.

First, a generative NLG model is used to create short human-like dialogues about the news content presented to the end users. Second, SA is applied to gather the polarity of the user utterances (we employ the supervised algorithm in [4] based on a dataset that was annotated manually). This introduces empathy to foster user engagement and allows annotating contextual data. In the following subsections we detail the building blocks, some of which are improved versions of those in [4]. All design decisions were consulted with gerontology experts and end users of AFAGA. This took six months.

### 3.1. News Broadcast Service

Elderly people spend much of their leisure time with traditional media, such as radio and television broadcasts, often as “background voices” [4,7]. It has been shown that this alleviates their solitude [8]. This observation led us to select a newscaster as the entertainment model of our solution. In particular, we chose the following topics of interest for our target audience:

- Economy;
- Leisure;
- Means of transport;
- Pensions;
- Politics;
- Public and social services;
- Science;
- Society;
- Sports;
- Well-being.

**Figure 2:**
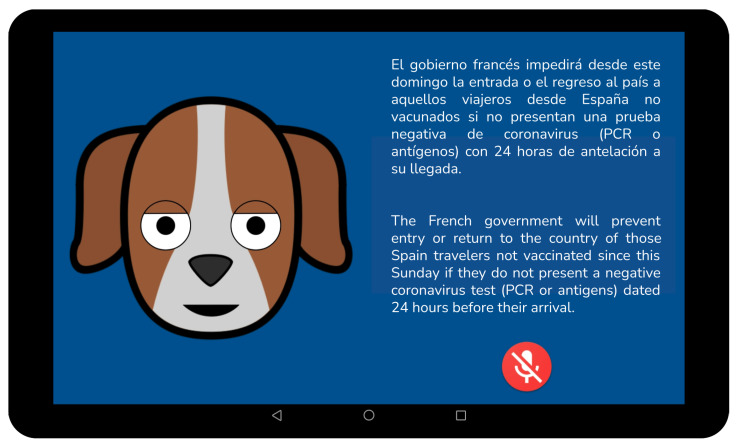
Lead paragraph sample.

By automatically browsing these categories in digital media, our conversational system presents the user with updated content and, between newscasts, it inserts short dialogues to gather user preferences. The content for this work was obtained through the “Radio y Televisión Española” (RTVE) API (available at https://www.rtve.es/api, accessed on 15 August 2021), whose output is presented as structured JSON and XML files. We simply apply an API filter based on the topic and date of publication. In addition to reading the newscast itself, the system presents the user with a summary consisting of the lead paragraph on the avatar screen (Figure 2, right).

### 3.2. Intelligent Dialogue Generation Service

#### 3.2.1. NLG Module for Flexible, Human-Like Dialogue Flows

We apply automatic text expansion from keywords to create complete, coherent and correct clauses in Spanish. For that purpose we use our three-stage hybrid NLG architecture (Text Planner, Sentence Planner and Realiser) [44,45] that relies on linguistic knowledge from our aLexiS lexicon and Spanish grammar, along with statistical data. This linguistic knowledge allows automatically adjusting gender, number, person and tense features to the input keywords. The statistical knowledge allows adding extra elements, such as prepositions, for the clauses to sound more natural.

**Figure 3:**
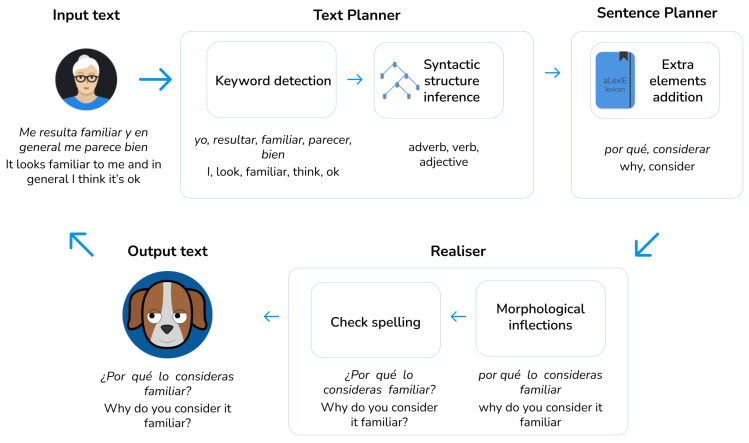
Text expansion scheme.

Figure 3 shows the text expansion procedure from the keywords obtained from the users. More in detail, the Text Planner infers the best syntactic structure from the grammar. Then, supported by the knowledge provided by the Sentence Planner, the NLG module adds the extra elements. Finally, the Realiser conducts morphological inflections and checks the spelling of the resulting clause. To illustrate this process, let us consider as a real example the user utterance *Me resulta familiar y en general me parece bien* ,“It looks familiar to me and in general I think it’s ok”. The first step is extracting the subject from the user’s clause, *yo*, “I”, along with the verb *resultar*, “look”, by relying on the information provided by aLexiS. Next, the NLG module obtains the opinion from the user’s keywords *bien*, “ok”, and *familiar*, “familiar”. Finally, the intelligent conversational assistant responds *¿Por qué lo consideras familiar?*, “Why do you consider it familiar?”.

The NLG module also exploits the knowledge provided by the SA module and, in case no keywords are extracted from the users’ responses, it selects template-based clauses according to the detected polarity. Specifically, to avoid monotonous utterances and ensure natural, varied and stimulating dialogue flows, the NLG module uses the polarity value provided by the SA module or its opposite based on a configurable likelihood.

A clause cache stores questions that are closely aligned to the news content. To create these questions we used again our aLexiS lexicon and the Name Entity Classification (NEC) module of Freeling (available at http://nlp.lsi.upc.edu/freeling/node/1, accessed on 15 August 2021) [46]. The latter allows the system to automatically identify people’s and organisations’ names as well as locations. To this end, our system can generate sentences with varied linguistic complexity levels. Low complexity examples are *¿Te suena el nombre de ENTIDAD?*, “Does the name ENTITY sound familiar to you?”, about people’s names; and *Cuéntame lo que más te ha gustado de ENTIDAD*, “Tell me what you liked the most about ENTITY?”, about places. High-complexity, more demanding sentences use noun, prepositional and verb clauses along with dates provided by Freeling from syntactic parsing based on linguistic dependencies. These elements are preceded by question words (*quién/quiénes*, “who”, and *qué*, “what”). *¿Qué ha explicado la ministra de industria?*, “What has the industry minister explained?”, is an example of a complex clause.

**Figure 4:**
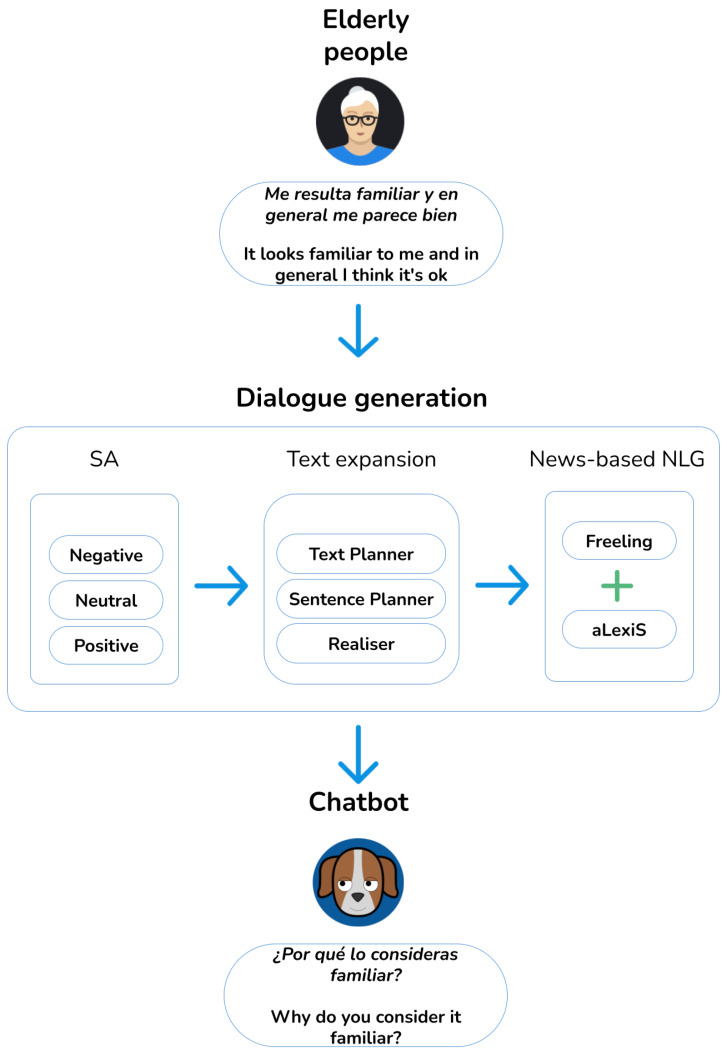
NLG module scheme taking into account the polarity of user responses.

Figure 4 shows the complete scheme of the NLG module taking into account the polarity of user responses.

#### 3.2.2. AIML-Based Human–Computer Interaction

As the core of the chatbot personality to complement the NLG module, we have created an ad hoc knowledge base using AIML. We remark that this information is solely employed to initiate the conversation with the end users in a controlled way, as small talk about daily activities and mood.

**Figure 5:**
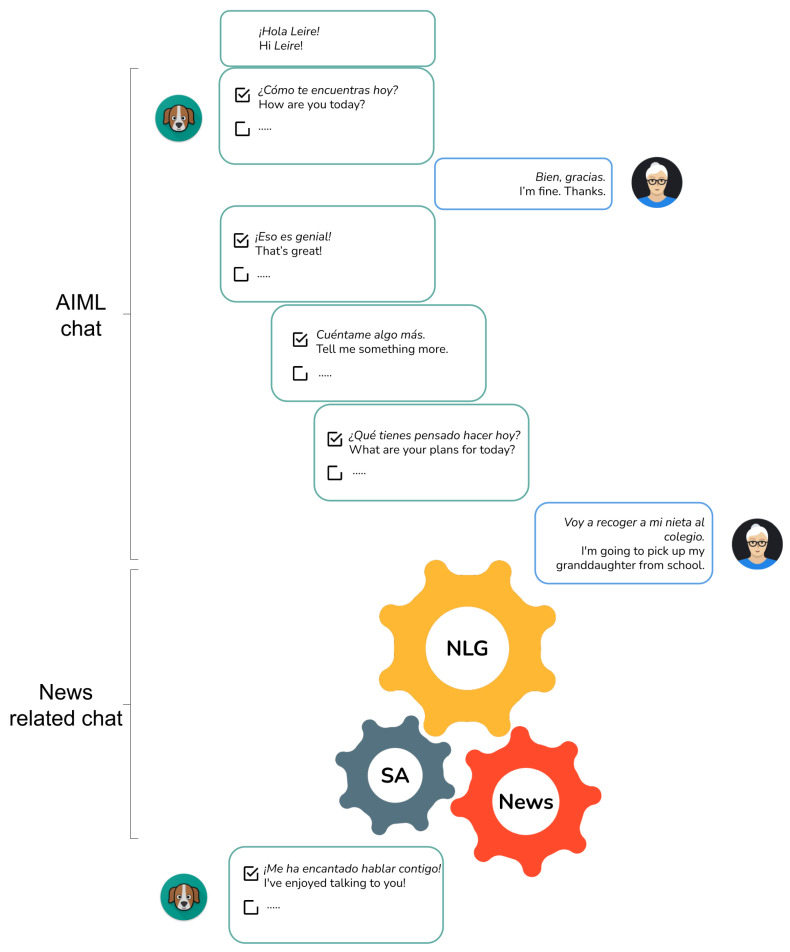
Dialogue flow scheme.

Figure 5 shows the dialogue flow, with examples of exchanges with the user in the initial dialogue (before the newscasts), based on templates. As in the case of the dialogue stages connecting newscasts, users’ utterances are expected to exhibit a certain polarity load with keywords such as *mal*, “bad” (negative meaning) and *bien*, “fine” (positive meaning). Thus, the chatbot can continue the conversation while taking into account the polarities in user answers.

To reduce frustration in the case of misunderstanding, the chatbot asks for clarifications instead of continuing talking. Even though we follow the recommendation to keep the interactions short to engage the users, by combining AIML-based utterances with those generated by the NLG module and the information provided by the SA module, we improve the degrees of freedom of user expressions.

### 3.3. Abstraction Assessment Module

We obtained the semantic classification of the relevant words in the news content (adjectives, adverbs, nouns and verbs) with the Multilingual Central Repository tool (MCR, available at http://adimen.si.ehu.es/web/MCR, accessed on 15 August 2021) [47]. We used Adimen SUMO, WordNet Domains and Top Ontology hierarchies for noun and verb elements, and only the latter for adjectives and adverbs. From the MCR tool we extracted holonyms, hypernyms, hyponyms, meronyms, related data (for nouns and verbs) and synonyms (for adjectives and adverbs).

We define an abstraction similarity metric varying between 0 (content not characterised at all) and 1 (perfectly characterised content) for each word element. Then, the overall similarity score sim for a response is computed as follows:

(1) $$\begin{array}{c} sim=0.8\frac{\sum_{i=1}^{N_{noun}}noun_{i}^{*}+\sum_{i=1}^{N_{verb}}verb_{i}^{*}}{N_{noun}+N_{verb}}\\ +0.2\frac{\sum_{i=1}^{N_{adj}}adj_{i}^{*}+\sum_{i=1}^{N_{adv}}adv_{i}^{*}}{N_{adj}+N_{adv}} \end{array}$$

where we weigh nouns and verbs categories as more relevant than adjectives and adverbs as suggested in [48], and:

- $N_{x}$ is the amount of words that belong to certain lexical category *x* in a gold standard consisting of the keywords that young, fully digital users employ to characterise the content.
- We evaluate a score *s* between each word in category *x* from the answer in the gold standard and all words in category *x* from the user’s response and select the highest value, $x_{i}^{*}$ by adapting the approach in [49]:

(2) $$\begin{array}{c} s\left(word_{1},word_{2}\right)=\left(1-\gamma \right)\alpha_{s}\beta^{d(word_{1},word_{2})}+\gamma , \end{array}$$

where:

- $\alpha_{s}=0.9$ if the words are synonyms, 0.85 otherwise.
- $d(word_{1},word_{2})=0$ if the words are holonyms, hypernyms, hyponyms, meronyms, synonyms or they are related to the same hierarchy category. Otherwise, $d(word_{1},word_{2})$ is WordNet’s shortest path between $word_{1}$ and $word_{2}$.
- β = 0.7 is a depth factor to control the score in terms of the distance between two words.
- γ is a correction factor to increase the score for words that belong to the same WordNet domain category or share the same stem. Its value is set as follows:
  - γ=0.25 if the words belong to the same WordNet domain category.
  - γ=0.5 for all terms sharing the same stem that are not synonyms and receive a score of 0.85 or less.
  - γ=0 otherwise.

We also consider numerical values in the news content. In this case, we save all possible rounded values of a number by dividing it by powers of 10. A score of 0.7 is assigned to user responses in case they include one of those coherent options. Additionally, if the user response includes modifiers such as “over” and “under” that are also coherent, the similarity score is increased to 0.9. Take the clause “167 litres per square metre have fallen in the city of Vigo” as an example. If the user’ response is “160 litres”, then sim=0.7. In case the answer is “under 200 litres”, then sim=0.9.

Therefore, the similarity metric allows evaluating how close is an answer to one of the control questions in the short dialogues to the characterisation of a content as a set of keywords in the gold standard. It permits assessing the abstraction skills of the elderly people in our tests, whose sentiment is also automatically detected from their answers.

### 3.4. Video and Audio Interfaces

The chatbot can be activated using video sensors through facial recognition. To this end, we used the popular OpenCV library (available at https://opencv.org, accessed on 15 August 2021) and an eye-sensing dataset (available at https://github.com/opencv/opencv/blob/master/data/lbpcascades/lbpcascade_frontalface.xml, accessed on 15 August 2021) to train the model. Audio sensors can also be used, that is, the chatbot activates itself automatically through voice commands. All subsequent interactions with the intelligent conversational system are vocal due to the good acceptance of this interface by the target users [50] for a more engaging experience [51]. To perform text-to-speech (TTS) and speech-to-text (STT) conversions we used the Google Voice Android Software Development Kit (SDK) in Spanish (available at https://developer.android.com/reference/android/speech/SpeechRecognizer, accessed on 15 August 2021).

Besides following widely accepted accessibility standards, we paid special attention to the requirements of users with hearing and vision impairments, by keeping the graphic appearance of the system simple and clear. This included a full-screen mode with large capitalised font and icon sizes, strong colour contrast and adequate volume settings.

**Figure 6:**
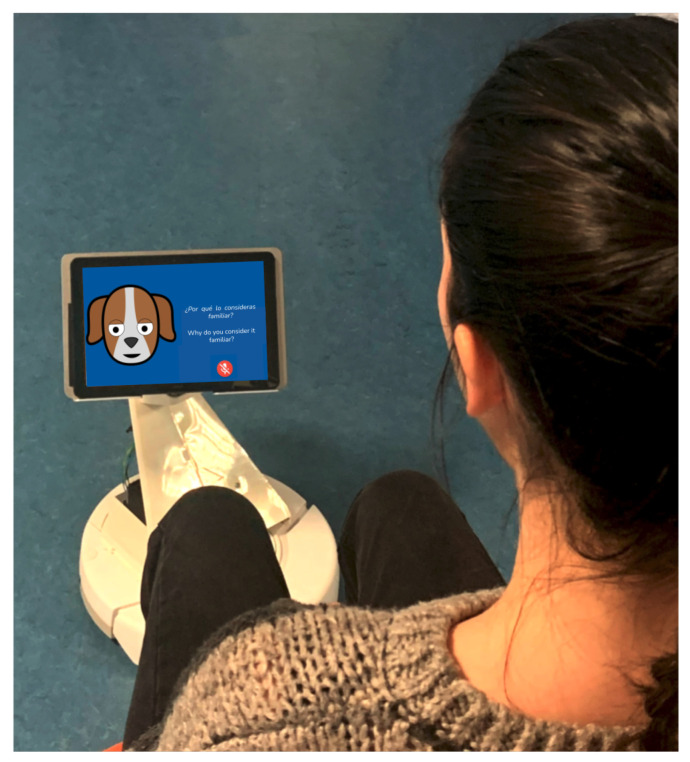
System appearance. The muted microphone under the text to the right of the avatar indicates the chatbot’s turn to talk.

Figure 6 shows the appearance of a system prototype on a mobile platform.

### 3.5. Home Automation Hub

Our intelligent conversational assistant can act as an automatic home hub by taking advantage of the knowledge extracted from man–chatbot interactions, thus helping to transform the user home into a smart and resourceful ecosystem of home devices and wearables.

For this purpose we exploit the WeMo solutions (available at https://www.wemo.com, accessed on 15 August 2021) by the market leader Belkin due to their reported extensibility and compatibility. WeMo devices are already compatible with conversational assistants such as Alexa from Amazon. They support several communication protocols, such as Wi-Fi, Bluetooth and ZigBee. Available actuators, including light bulbs, can also be controlled with our system with UPnP commands. The built-in intelligence of our assistant can be especially advantageous in terms of safety for the target users, elderly people who may live alone, by filtering actions such as inadequate ambient temperature settings. Our expert system can also control security systems and activate emergency calls based on its knowledge.

## 4. Experimental Results and Discussion

In this section we evaluate our system. First, we present experimental results of the SA module (Section 4.1), which are essential to automatically annotate user responses for subsequent analyses. Then, we analyse the data gathered by the smart conversational system in two experiments: (1) The relation between user interest and abstraction skills using our own cognitive metric (Section 4.2), and (2) the detection with ML of discomfort when accessing entertainment content (Section 4.3).

In both experiments, data were automatically obtained from sessions with senior end users that interacted with the newscaster chatbot. Then, data were transferred to a server for further analysis. The data gathering process was supervised by gerontology experts from AFAGA.

More in detail, in experiment 1 above we studied the relation between sentiment scores (which we consider to be indicative of the level of interest) and the sim measure of abstraction capability (see Section 3.3). Then, in experiment 2 we studied the performance of the system to detect discomfort of end users with ML techniques. In this second experiment we exploited knowledge from the SA module and the sim measure of the abstraction assessment module, along with the users’ interactions during the news-related dialogue stage.

For the experimental tests, we deployed the NLG and SA services on a server with 64 GB RAM and 12 cores. The user interface was presented on a handheld Huawei MediaPad T5 tablet. There were 20 participants in the tests, with equal representations of women and men, 75.55 ± 7.07 years old (average ± standard deviation), distributed as follows regarding technological background and discomfort:

- Nine of the users had technological background.
- Eight of the users experienced discomfort to a noticeable extent (were confused/stressed/unfocused during the tests).

In order to annotate discomfort, when the session ended, the users were asked to fill a survey to gather the following information (yes/no answers were expected):

- Confusion;
- Stress;
- Concentration.

Furthermore, complementing the responses in the connecting dialogues, after each newscast the users were asked to provide the keywords they would use to find similar content in a source. The participants listened to 4.4 newscasts on average. Finally, they rated the experience on a five-level Likert scale regarding:

- Satisfaction;
- Amazement;
- Human–computer interaction naturalness (compared to a human interaction).

User ratings were coherent with the comfort survey.

### 4.1. SA Module Performance

The SA module [4] estimates the polarity among three possible levels: negative, neutral and positive. We tried different ML algorithms using the implementations in the Scikit-Learn Python library. These specific choices were selected based on their good performance in similar problems [4,52,53,54]:

- Decision Tree (DT) (available at https://scikit-learn.org/stable/modules/tree.html, accessed on 15 August 2021);
- Gradient Descent (GD) (available at https://scikit-learn.org/stable/modules/sgd.html, accessed on 15 August 2021);
- Random Forest (RF) (available at https://scikit-learn.org/stable/modules/generated/sklearn.ensemble.RandomForestClassifier.html, accessed on 15 August 2021);
- Support Vector Classification (SVC) (available at https://scikit-learn.org/stable/modules/generated/sklearn.svm.SVC.html, accessed on 15 August 2021).

Table 1 shows their performance with 10-fold cross validation. The best algorithm for our application was Decision Tree with 80.7% precision, 79.8% recall, 80.0% accuracy and 79.7% F1 score.

### 4.2. Relation between Interest and Augmentation of Abstraction Capabilities

As previously said, this analysis was performed using both the news-related chat (Figure 5, centre) and, within it, the keywords with which the target users described the news presented by our chatbot. An external annotator with technological proficiency and cognition expertise manually established the gold standard for such keywords.

For each newscast we first divided the dialogue stage into negative and positive polarity classes, which we assumed to represent uninteresting and interesting digital content for the elders, respectively. We then computed the sim metric in Section 3.3 using their responses.

Consideration should be given to the correlation between the polarity and the sim metric, 0.47. This relatively high value demonstrates that polarities are adequate to annotate interest, since a higher abstraction is indicative of a higher concentration and, thus, of interest in the content. In other words, the sim metric is higher in case of positiveness. Accordingly, Table 2 presents average sim values by polarity. Note the 20% increase, from 0.333 to 0.537, when the users were presented with digital content of their interest.

Table 3 presents a further subdivision of the results by grouping the users into those that were concentrated, focused and relaxed during the tests and had some technological background (group 0) and those that were confused or stressed during the tests and were not keen on technology (group 1). We consider that the people in this second group experienced discomfort. The trend by polarity, regardless of group, did not change. For both polarities, sim results were always lower for group 1, although this effect was more evident in negative users, which we assume to be less interested (0.280 versus 0.374 for negative polarity and 0.528 versus 0.549 for positive polarity).

### 4.3. Detection of Possible Discomfort with ML

For detecting possible discomfort in the target users with ML techniques, user utterances were first pre-processed to avoid noisy and irrelevant information to ensure the quality of the data that enter the ML classification module. The pre-processing stage was composed of the following steps:

- Filtering. It removes stop words, such as prepositions and conjunctions, using the NLTK Python module (available at https://www.nltk.org, accessed on 15 August 2021). In addition, to detect and discard words with a low semantic load, only words with at least four characters were kept. Moreover, non-Spanish words were discarded using the Enchant Python module (available at https://pypi.org/project/pyenchant, accessed on 15 August 2021).
- Spelling correction. In case the Google Voice Android Software Development Kit voice-to-text conversion failed, we used the Enchant Python module and the word distance dataset by “Real Academia Española de la Lengua” (Royal Spanish Academy of the Language) to correct the transcription (available at http://corpus.rae.es/lfrecuencias.html, accessed on 15 August 2021).
- Word lemmatisation. User utterances were first tokenised and then lemmatised to reduce the variability of the word space using Freeling.

The input features were polarity level, sim metric and word *n*-grams. We generated these word *n*-grams of users’ utterances from the newscast dialogue stages. We used CountVectorizer (available at https://scikit-learn.org/stable/modules/generated/sklearn.feature_extraction.text.CountVectorizer.html, accessed on 15 August 2021). In this case, the best results were obtained with min_df = 0.001, max_df = 0.5 and ngram_range = (1,1).

For the classification task we employed the implementations of the DT, GD, RF and SVC algorithms from the Scikit-Learn Python library. Listings 1–4 provide the configurations for this set of ML models, highlighting our selections in boldface. These configurations were obtained with GridSearchCV (available at https://scikit-learn.org/stable/modules/generated/sklearn.model_selection.GridSearchCV.html, accessed on 15 August 2021).

Listing 1 **Configuration parameters for the DT classifier, best settings in boldface.**

class_weight: [balanced, **None**],

criterion: [**entropy**, gini],

max_depth: [1, 2, 3, 4, 5, 6, 7, **8**],

max_features: [auto, log2, **sqrt**],

min_samples_leaf: [0.005, **0.01**, 0.05, 0.1],

min_samples_split: [**0.005**, 0.01, 0.05, 0.1],

splitter: [best, **random**]

Listing 2 **Configuration parameters for the GD classifier, best settings in boldface.**

learning_rate: [0.001, **0.15**],

max_depth: [2, **6**],

min_samples_leaf: [0.005, **0.1**],

min_samples_split: [0.005, **0.1**],

n_estimators: [150, **200**, 2000],

subsample: [0.8, **0.9**]

Listing 3 **Configuration parameters for the RF classifier, best settings in boldface.**

criterion: [**entropy**, gini],

max_depth: [2, 10, **50**],

min_samples_leaf: [**0.0001**, 0.001, 0.1, 0.5],

min_samples_split: [**0.1**, 0.10, 0.5],

n_estimators: [**180**, 400, 600, 800, 1000, 1200, 2000]

Listing 4 **Configuration parameters for the SVC classifier, best settings in boldface.**

C: [0.0001, 0.0005, 0.001, 0.01, **0.1**],

loss: [hinge, **squared_hinge**],

max_iter: [500, 1000, **1500**],

multi_class: [crammer_singer, **ovr**],

penalty: [l1, **l2**],

tol: [**0.000000001**, 0.000001, 0.001, 0.1]

Table 4 shows the results of discomfort detection for the performance metrics under study: precision, recall, F1 score and accuracy, and for diverse combinations of the features in the model in growing complexity. Test #1 set a baseline only defined by the sim metric and polarity. Even though the recall value for the SVC classifier was satisfactory, precision and accuracy were low in this case. In test #2 we added *n*-gram features to the models, which enhanced the results of RF and GD, although the precisions were under 70% in all cases. For further refinement, in test #3 we applied feature selection with SelectPercentile. Finally, in test #4 with hyperparameter optimisation we finally achieved satisfactory performance levels above 80% with all the metrics. Based on the results obtained, we selected SVC as the best ML model with the configuration in test #4.

## 5. Conclusions

In [4] we proposed an entertainment chatbot for the digital inclusion of elderly people that enhances their abstraction capabilities to find content of their interest. We have evolved it into a smart tele-assistance system that can interact with home sensors and actuators.

In this work we described its improvements and, based on them, some analyses that demonstrate that the SA of user responses is a good approximation of user interest, to extract keywords to better personalise content with the automatic abstraction capabilities of the system. Then, we showed that the chatbot can detect discomfort in the target users with over 80% precision, recall, F1 score and accuracy.

As future work we plan to evaluate the positive feedback to user cognition due to the automatic abstraction capabilities. That is, the ability of the system to indirectly “teach” the user to provide better characterisations of the content of interest in his/her responses.

## Figures and Tables

**Table 1:** Performance analysis of the ML algorithms for polarity detection in user responses.

Model	Precision	Recall	F1	Accuracy
DT	**0.807**	**0.798**	**0.797**	**0.800**
GD	0.762	0.742	0.741	0.745
RF	0.748	0.733	0.730	0.735
SVC	0.800	0.788	0.786	0.788

**Table 2:** sim results grouped by SA result.

Polarity	sim Value
Negative	0.333
Positive	0.537

**Table 3:** sim results by polarity value and user group.

Polarity	Group	sim Value
Negative	0	0.374
	1	0.280
Positive	0	0.549
	1	0.528

**Table 4:** Accuracy of different ML models, for diverse combinations of features, to classify users between groups 0 and 1, and precision and recall values for group 1.

#	Features	Model	Precision	Recall	F1	Accuracy
1	sim + polarity	DT	0.625	0.600	0.612	0.563
		GD	0.596	0.560	0.577	0.529
		RF	**0.638**	0.600	0.619	0.575
		SVC	0.614	**0.860**	**0.717**	**0.609**
2	sim + polarity + *n*-grams	DT	**0.681**	0.640	0.660	**0.621**
		GD	0.635	0.660	0.647	0.586
		RF	0.580	**0.800**	**0.672**	0.552
		SVC	0.542	0.520	0.531	0.471
3	sim + polarity + *n*-grams with feature selection	DT	0.600	0.600	0.600	0.540
		GD	0.698	0.740	0.718	0.667
		RF	0.690	**0.980**	**0.810**	**0.736**
		SVC	**0.745**	0.760	0.752	0.713
4	sim + polarity + *n*-grams with feature/hyperparameter	DT	0.629	0.880	0.733	0.632
		GD	0.706	0.720	0.713	0.667
		RF	0.685	**1.00**	0.813	0.736
		SVC	**0.824**	0.840	**0.832**	**0.805**

## Data Availability

Data will be made available to other researchers upon request and authorisation by AFAGA.
